# The Anti-Nociceptive Effects of Nicotine in Humans: A Systematic Review and Meta-Analysis

**DOI:** 10.3390/ph16121665

**Published:** 2023-11-30

**Authors:** Yujia Luo, Yating Yang, Carl Schneider, Thomas Balle

**Affiliations:** 1 Sydney Pharmacy School, Faculty of Medicine and Health, The University of Sydney, Sydney, NSW 2006, Australia; yujialuo@zju.edu.cn (Y.L.);; 2 Brain and Mind Centre, The University of Sydney, 94 Mallet Street, Camperdown, NSW 2050, Australia

**Keywords:** pain, nicotine, tobacco, analgesic, analgesia, systemic review, meta-analysis

## Abstract

Background: Pain can have a serious impact on a patient’s physical, mental, and social health, often causing their quality of life to decline. Various nicotine dosage forms, such as nicotine patches and nasal spray, have been developed and used as analgesics in clinical settings. However, there is controversy over the anti-nociceptive effects of nicotine among different clinical trials. The purpose of this meta-analysis is to quantify the analgesic effect of nicotine patches, nicotine nasal spray, and tobacco smoking on pain in humans. Methods: Relevant articles published in English prior to July 2023 were identified using the PubMed, Cochrane Library, and Embase online databases in accordance with PRISMA (2020) guidelines. Two reviewers independently screened and selected studies, extracted data, and assessed the quality of the included studies using version 2 of the Cochrane risk-of-bias tool for randomized trials (RoB 2). RStudio was used for data synthesis, heterogeneity assessment, sensitivity analysis, publication bias assessment, trim-and-fill analyses, and generating forest plots. Results: Sixteen eligible articles, including k = 5 studies of pain tolerance (*n* = 210), k = 5 studies of pain threshold (*n* = 210), and k = 12 studies of pain scores (N = 1249), were included for meta-analysis. Meta-analytic integration for pain threshold (Hedges’ g = 0.28, 95% CI = 0–0.55, Z = 1.99, *p* = 0.05) and pain tolerance (Hedges’ g = 0.32, 95% CI = 0.05–0.59, Z = 2.30, *p* = 0.02) revealed that nicotine administered via tobacco smoke generated acute analgesic effects to thermal stimuli. Meta-analytic integration for pain scores revealed that nicotine had a weak anti-nociceptive effect on postoperative pain of −0.37 (95% CI = −0.77 to 0.03, Z = −1.80) but with no statistical significance (*p* = 0.07). In addition, a limited number of included studies revealed that long-term smoking produced hyperalgesia that may be characterized as small to medium in magnitude (Hedges’ g = 0.37, 95% CI = 0.29–0.64, Z = 5.33, *p* < 0.01). Conclusion: These results help to clarify the mixed outcomes of trials and may ultimately inform the treatment of pain. We observed that acute nicotine administration prolonged the laboratory-induced pain threshold and tolerance time and may mildly relieve postoperative pain. In addition, long-term tobacco smoking may have a nociceptive effect on different types of chronic pain. More research is needed to determine the anti-nociceptive effects of nicotine in humans, and to understand the optimal timing, dose, and method of delivery of nicotine.

## 1. Introduction

Pain is associated with numerous health problems and is a common reason for seeking medical care. It is a major cause of diseases and disabilities [1,2] and is listed as the fifth vital sign of mankind. The economic burden of pain for society and the health care system is unquestionably substantial. In July 2020, the International Association for the Study of Pain (IASP) revised the definition of ‘pain’ as follows: “An unpleasant sensory and emotional experience associated with, or resembling that associated with, actual or potential tissue damage.” It is well known that pain is a significant contributor to a lower level of quality of life (QOL) in humans. This negative impact has been found to span patients in all age groups and is associated with all types and sources of pain studied. For centuries, the medical community has been exploring the efficacy of various substances and therapeutic approaches to alleviating pain. Effective analgesic therapy has been shown to improve quality of life by relieving pain.

As far back as the sixteenth century, it was known that tobacco could provide relief from pain, such as that caused by syphilis. However, it was not until the late twentieth century that the analgesic properties of nicotine, the primary component of tobacco, were systematically researched and clinically applied [3]. Nicotine occupies a pivotal role in the intricate landscape of pain-related pathophysiology. Its influence on pain perception is multifaceted and involves a range of processes. The acute analgesic effects of nicotine are orchestrated through the activation of nicotinic acetylcholine receptors (nAChRs) in both the central and peripheral nervous systems [4,5]. These nAChRs serve as critical gatekeepers, influencing the release of neurotransmitters like noradrenaline, endogenous opioids, dopamine, and others. These neurotransmitters, in turn, have a dual role: they can either set in motion the descending pain modulatory pathway or stifle the incoming nociceptive signals to the spinal dorsal horn [6,7]. Nowadays, different forms of nicotine delivery, like nicotine patches and nasal spray, have been developed and employed as analgesics in clinical settings [8,9].

Upon careful review of the relevant empirical literature, it becomes evident that there is controversy surrounding the anti-nociceptive (pain-relieving) effects of nicotine, as observed in various clinical trials. For instance, five clinical studies involving laboratory-induced pain found that short-term application increased pain thresholds and pain tolerance to cold percussion tests in healthy volunteers [10,11,12,13,14]. Three clinical studies documented the effectiveness of both nicotine patches and nicotine nasal sprays in alleviating postoperative pain [8,15,16]. In contrast, six clinical studies revealed that the anti-nociceptive effect of nicotine patches and nicotine nasal spray on postoperative pain was less apparent [9,17,18,19,20,21]. Additionally, some studies have indicated that long-term smoking may exacerbate chronic pain conditions [22,23]. Consequently, despite decades of research, the extent of nicotine’s analgesic effects in humans remains uncertain.

In this context, the present systematic review with meta-analysis aims to determine the analgesic effect of nicotine patches, nicotine nasal spray, and tobacco smoking on pain in humans.

## 2. Materials and Methods

### 2.1. Search Strategy

Human studies that included participants of both sexes and examined the effects of nicotine were considered. There was no restriction on age, the severity of the pain (mild, moderate, or severe), or the location of the surgical wound. All prospective randomized controlled trials (RCTs) or self-controlled studies published prior to July 2023 were collected using the PubMed, Cochrane Library, and Embase online databases in accordance with PRISMA (2020) guidelines. Searches through PubMed were conducted using the MeSH terms for “pain” or keywords for “analgesi*” in combination with the MeSH terms for “nicotine” or keywords for “tobacco/cigarette”. Searches through Cochrane Library and Embase were conducted using the major search term “pain” or “analgesi*” in combination with the major search terms “nicotine”, “tobacco”, or “cigarette”. The full search strategies for these databases are listed below:

(1) Search strategy via PubMed:

#1 (‘Pain’[Mesh]) OR (‘analgesia’[Title/Abstract]) OR (‘analgesic’[Title/Abstract])

#2 (‘Nicotine’[Mesh]) OR (‘tobacco’[Title/Abstract]) OR (‘cigarette’[Title/Abstract])

#1 AND #2

(2) Search strategy via Cochrane Library:

#1 (‘pain’):ti,ab,kw OR (‘analgesia’):ti,ab,kw OR (‘analgesic’):ti,ab,kw

#2 (‘nicotine’):ti,ab,kw OR (‘tobacco’):ti,ab,kw OR (‘cigarette’):ti,ab,kw

#3 #1 AND #2

(3) Search strategy via Embase:

#1 ‘pain’:ab,ti OR ‘analgesia’:ab,ti OR ‘analgesic’:ab,ti

#2 ‘nicotine’:ab,ti OR ‘tobacco’:ab,ti OR ‘cigarette’:ab,ti

#3 #1 AND #2 AND [article]/lim AND [humans]/lim AND [embase]/lim

### 2.2. Inclusion Criteria

To be included in this study, trials were required to meet the following criteria: (1) the pain degree of the participants was quantified via pain scores, such as the numerical rating scale (NRS) or visual analogue scale (VAS), or pain threshold and/or tolerance time; (2) sample was comprised of human participants; (3) the study was conducted as a randomized controlled trial or as self-controlled studies; (4) nicotine administered to patients experiencing pain or nicotine administered in combination with the utilization of an experimental/laboratory method of pain induction was compared with non-nicotine controls; (5) studies used either a between-subject or within-subject design.

This systematic review was registered with the International Prospective Register of Systematic Reviews (https://www.crd.york.ac.uk/PROSPERO, accessed on 20 May 2021). The registration number is PROSPERO CRD42021250138. As no identifying information about personal details is shown in this paper, ethical approval and informed consent were not needed.

### 2.3. Data Extraction and Risk of Bias

Data were extracted and coded by two independent reviewers (YL and YY). Protocol and treatment outcome data were extracted from the studies included in this systemic review and meta-analysis. The information extracted included the author, publication year, pain induction method (e.g., thermal, surgical, or other), efficacy measures (e.g., pain threshold, pain tolerance, or pain scores), treatment regimen (e.g., nicotine patches, nicotine nasal spray, or tobacco smoking), control condition (no nicotine administration), study design (between-/within-subject design) and sample size. To pool the results in a consistent format, the sample mean and standard deviations were estimated from the sample size, median, mid-range, and/or mid-quartile range according to the previously published transformation methodology [24] and the “Cochrane Handbook for Systematic Reviews of Interventions” (https://training.cochrane.org/handbook, accessed on 20 May 2021). We extracted the following data: author, published year, intervention, measurement of pain, type of study design, and number of patients in each patient group. The risk of bias in each study was evaluated according to the Newcastle–Ottawa Scale (NOS) by two reviewers (YL and YY), who were blinded to each other’s assessment. Any disputes were arbitrated by consulting two experienced reviewers (CS and TB).

### 2.4. Quality Assessment

The methodological quality, including the risk of bias of the included studies, was evaluated using version 2 of the Cochrane risk-of-bias tool for randomized trials (RoB 2). Each trial was reviewed by two reviewers (YL and YY), and differences were resolved by consensus or referral to two reviewers (CS and TB). Trials scoring 1–2 points were considered to be of low quality and were excluded from this systemic review and meta-analysis.

### 2.5. Data Analysis

We performed a meta-analysis using RStudio. To conduct meta-analytic integrations, we employed RStudio packages, including ‘meta’, ‘metafor’, ‘readr’, ‘Matrix’, and ‘esc’. Study weights were assigned according to the inverse variance method, and calculations were based on a fixed-effect model or random-effects model (depending on the heterogeneity of the outcome). An alpha value of 0.05 was adopted. Effect sizes were calculated using Hedge’s g due to the sample size for each individual study being relatively small. Statistical heterogeneity among the studies was tested using the Cochran Q test, and inconsistency was assessed using the I^2^ index. Briefly, the I^2^ index reflects the extent of heterogeneity (percentage of the total variability) across effect sizes, with I^2^ values of 25%, 50%, and 75% corresponding to low, moderate, and high levels of heterogeneity, respectively [25]. In cases where primary analyses unveiled indications of heterogeneity, we carried out sensitivity analyses. If homogeneity was not observed, the fixed effect model was utilized to amalgamate dichotomous data. Alternatively, when heterogeneity was detected, we opted for the random effects model. To examine potential publication bias, we utilized Egger’s test and/or a visually evaluated funnel plot. In scenarios where primary analyses suggested the presence of publication bias, we performed a trim-and-fill analysis.

## 3. Results

### 3.1. Study Selection and Characteristics of the Study Samples

**Figure 1:**
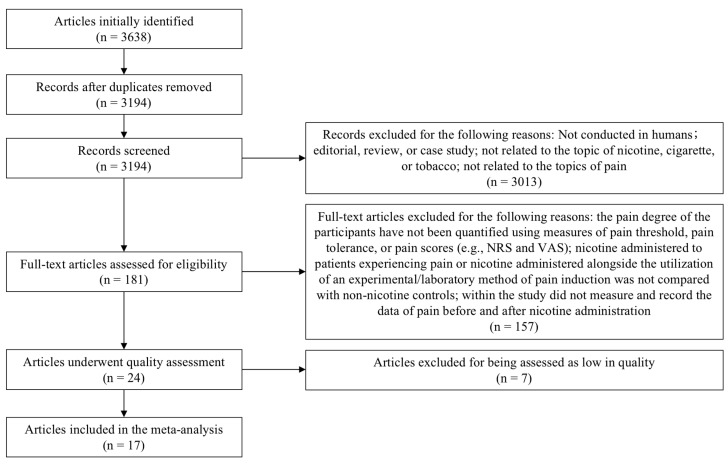
PRISMA diagram of study selection.

A flowchart of our approach is presented in Figure 1. Of the 3638 articles identified as potentially relevant via the initial searches, 444 were found to be duplicates, and the remaining 3194 were screened for inclusion. A further 181 were deemed potentially eligible and worthy of full-text review. Of these, 157 articles were excluded for not meeting the inclusion criteria. The remaining 24 articles were assessed according to RoB2, and 7 of them were excluded due to the papers not presenting the outcomes clearly or being assessed as low in quality. In this systematic review with meta-analysis, a total of 17 studies were found to be eligible, including 5 studies of pain tolerance (*n* = 210), 5 studies of pain threshold (*n* = 210), and 12 studies of pain scores (*n* = 1249) [8,9,10,11,12,13,14,15,16,17,18,19,20,21,22,23,24]. The descriptive information and risk of bias assessment for each of these 17 trials are presented in Table 1.

### 3.2. Effect of Nicotine on Pain Threshold

**Figure 2:**
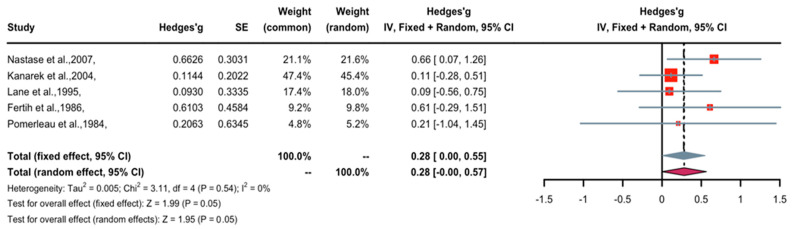
Effective size, confidential interval, and forest plot of five studies associating nicotine with pain threshold time [10,11,12,13,14].

A forest plot of Hedge’s g effect size and 95% confidential interval (CI) for a change in pain threshold as an outcome of nicotine application is presented in Figure 2. The fixed effects model was used as no heterogeneity was observed in the included studies. Three studies (weight 69.6%) found nicotine to have weak anti-nociceptive effects, two studies (weight 30.3%) revealed significant anti-nociceptive effects, and no studies showed contrasting data (nociceptive) regarding the effects of nicotine. Overall, the Hedges’ g effect size was 0.28 (95% CI = 0–0.55, Z = 1.99, *p* = 0.05), indicating that nicotine had an anti-nociceptive effect on pain threshold that may be characterized as small to medium in magnitude. The trim-and-fill method was applied to remove publication bias. The five studies were imputed to create symmetry in the pain threshold funnel plot, and after doing so, the Hedge’s g effect size was reduced to non-significance (0.18, 95% CI = −0.11–0.47). This suggests that publication bias may be influencing the outcome of nicotine’s effect on prolonging pain threshold time.

### 3.3. Effect of Nicotine on Pain Tolerance

**Figure 3:**
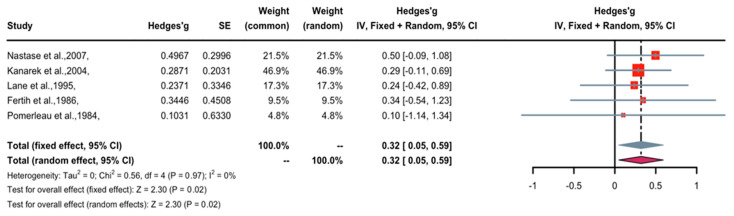
Effective size, confidential interval, and forest plot of five studies associating nicotine with pain tolerance time [10,11,12,13,14].

A forest plot of Hedge’s g effect size and 95% CI for a change in pain tolerance as an outcome of nicotine application is presented in Figure 3. The fixed-effects model was used as no heterogeneity was observed in the included studies. One study (weight 4.8%) found nicotine to have weak anti-nociceptive effects, three studies (weight 73.7%) revealed medium anti-nociceptive effects, one study (weight 21.5%) demonstrated significant anti-nociceptive effects, and no studies found contrasting (nociceptive) effects. Overall, the Hedges’ g effect size was 0.32 (95% CI = 0.05 to 0.59, Z = 2.30, *p* = 0.02), indicating that nicotine had an anti-nociceptive effect on pain tolerance that may be characterized as small to medium in magnitude. The trim-and-fill method was applied to remove publication bias. The five studies were imputed to create symmetry in the pain threshold funnel plot, and after doing so, the Hedge’s g effect size was raised from 0.32 to 0.33 (95% CI = 0.07 to 0.60). This suggests that publication bias has little or no effect on the outcomes of nicotine’s effect on prolonging pain tolerance time.

### 3.4. Effect of Short-Term Nicotine Application in Patients with Postoperative Pain

**Figure 4:**
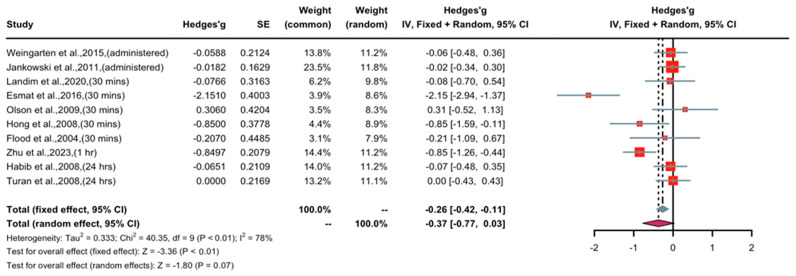
Effective size, confidential interval, and forest plot of nine studies associating short-term nicotine application with pain score outcomes [8,9,15,16,17,18,19,20,21,26].

**Figure 5:**
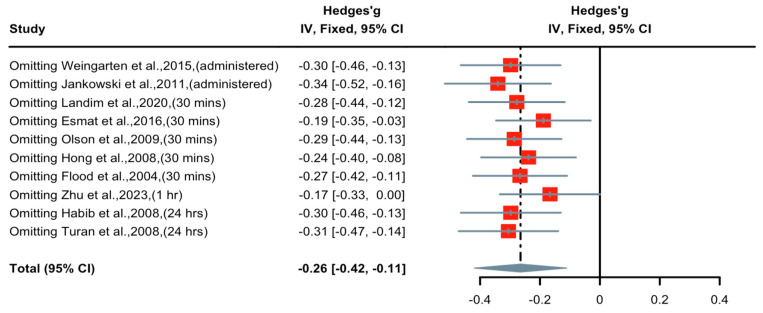
Effective size, confidential interval, and forest plot of nine studies associating short-term nicotine application with pain score outcomes after sensitivity analysis [8,9,15,16,17,18,19,20,21,26].

A forest plot of Hedge’s g effect size and 95% CI for a change in pain score (NRS or VAS) as an outcome of short-term nicotine application in postoperative pain is presented in Figure 4. The random effect model was used as a high level of heterogeneity (I^2^ = 78%) was observed in the included studies. Seven studies (weight 71.3%) found nicotine to have weak or no anti-nociceptive effects, two studies (weight 20.1%) revealed medium anti-nociceptive effects, and one study (weight 8.6%) demonstrated significant anti-nociceptive effects. Overall, the Hedges’ g effect size was −0.37 (95% CI = −0.77 to 0.03, Z = −1.80, *p* = 0.07), indicating that nicotine had an anti-nociceptive effect on postoperative pain that may be characterized as small in magnitude. Sensitivity analysis was applied to reduce the heterogeneity of the included studies. A forest plot of Hedge’s g effect size and 95% CI after sensitivity analysis for a change in pain score (NRS or VAS) as an outcome of short-term nicotine application in postoperative pain is presented in Figure 5. Hedge’s g effect size was reduced from −0.37 to −0.26 (95% CI = −0.42 to −0.11). There was no evidence of publication bias across pain score comparisons. This suggests that nicotine’s effect on relieving postoperative pain may be significant after adjusting for publication bias.

### 3.5. Effect of Long-Term Nicotine Application in Patients with Chronic Pain

**Figure 6:**
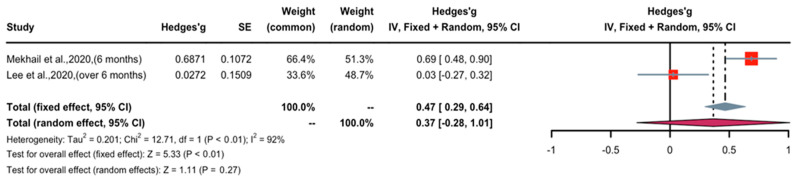
Effective size, confidential interval, and forest plot of two studies associating long-term nicotine exposure with pain score outcomes [22,23].

**Figure 7:**
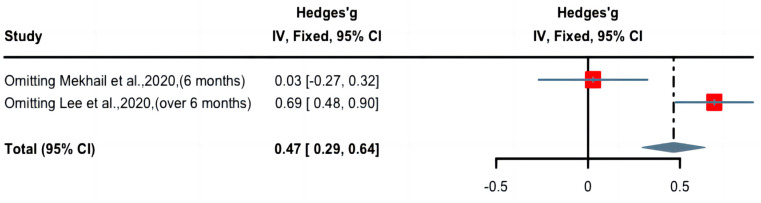
Effective size, confidential interval, and forest plot of two studies associating long-term nicotine exposure with pain score outcomes after sensitivity analysis [22,23].

A forest plot of Hedge’s g effect size and 95% CI for a change in pain score (NRS or VAS) as an outcome of long-term nicotine application in patients with chronic pain is presented in Figure 6. The random effect model was used as high levels of heterogeneity (I^2^ = 92%) were observed in the included studies. One study (weight 48.7%) found long-term nicotine application to have weak nociceptive effects, whereas one study (weight 51.3%) demonstrated significant nociceptive effects. Overall, the Hedges’ g effect size was 0.37 (CI: 0.29 to 0.64, Z = 5.33, *p* < 0.01), indicating that long-term nicotine administration had a nociceptive effect on chronic pain that may be characterized as small to medium in magnitude. Sensitivity analysis was applied to reduce the heterogeneity of the included studies. A forest plot of Hedge’s g effect size and 95% CI after sensitivity analysis for a change in pain score (NRS or VAS) as an outcome of long-term nicotine application in chronic pain is presented in Figure 7. Hedge’s g effect size was increased from 0.37 to 0.48 (95% CI = 0.29 to 0.64). There was no evidence of publication bias across pain score comparisons. This suggests that publication bias does not significantly affect the effect of long-term nicotine usage in attenuating chronic pain.

## 4. Discussion and Conclusions

### 4.1. Summary

Pain, a complex and multifaceted phenomenon, significantly impacts an individual’s overall wellbeing, affecting physical, mental, and social health. Limited data exist on the relationship between smoking and acute or chronic pain. Nicotine plays a vital role in pain-related processes. Despite providing short-term pain relief, prolonged nicotine exposure leads to tolerance and increased pain sensitivity due to receptor desensitization and neural changes [26]. Seyedsadeghi et al. indicated that there were no significant statistical disparities between the groups concerning pain intensity. They also reported nausea and vomiting across various postoperative time intervals in the nicotine group, and 24 in the placebo group were administered meperidine (*p* > 0.05) [27]. In the pursuit of pain management, various forms of nicotine administration, such as nicotine patches and nasal sprays, have been explored for their analgesic potential. Da Silva Barbirato et al. indicated that there is a potential for transdermal nicotine patches to provide analgesic benefits in surgical settings, although the quality of evidence is relatively low [28]. However, clinical trials have yielded mixed results, leading to debates over the anti-nociceptive effects of nicotine.

Our systemic review and meta-analysis, consisting of 16 eligible articles, provides valuable insights into the analgesic potential of nicotine. The presented analyses help clarify conflicting literature data and may ultimately help to inform the treatment of pain. Based on our meta-analyses, we observed that: (1) acute administration of nicotine through tobacco smoke demonstrated acute analgesic effects on thermal stimuli. This suggests that nicotine can raise pain thresholds and extend pain tolerance. These findings align with previous studies indicating that short-term nicotine exposure can increase pain thresholds and tolerance, especially in laboratory-induced pain scenarios. (2) The analysis of pain scores revealed a relatively weaker anti-nociceptive effect of nicotine on postoperative pain within 24 h compared with placebo, but with no statistical significance (*p* = 0.11). This highlights the complexity of nicotine’s analgesic properties, indicating that its effectiveness in managing postoperative pain may not be as robust as in laboratory-induced pain scenarios. (3) This study also touched upon the long-term effects of nicotine. It found that long-term tobacco smoking may contribute to hyperalgesia in chronic pain conditions, showing that the relationship between nicotine and pain management is not one-dimensional. This finding raises concerns about the consequences of chronic nicotine exposure and its potential to worsen chronic pain conditions.

In the realm of pain management, nicotine exhibits analgesic effects, particularly in the acute pain context. There is a need for further research that fine-tunes the administration of nicotine for acute pain scenarios, encompassing the determination of the ideal dosage, timing, and delivery method. Moreover, the intricate relationship between chronic pain and long-term tobacco smoking deserves thorough exploration to comprehend potential hyperalgesic effects. Future studies should delve into the mechanisms underlying this phenomenon, assess the impact of nicotine cessation on chronic pain patients, and explore strategies for alleviating this effect. As we seek to harness nicotine’s potential as an analgesic, it is imperative to investigate alternative delivery methods that mitigate the health risks associated with smoking, such as e-cigarettes or novel pharmaceutical formulations. Striking the right balance between pain relief and potential adverse effects hinges on determining the optimal nicotine dosage through extensive research. Additionally, one must consider the inter-individual variability in response to nicotine, with factors like genetics, smoking history, and comorbidities potentially influencing how individuals respond to nicotine as an analgesic.

### 4.2. Potential Mechanisms of Acute Nicotine-Induced Antinociception and Long-Term Hyperalgesia

Acetylcholine receptors (AChRs) can be divided into two major classes according to the response to different alkaloids: namely, nicotinic AChRs (nAChRs) and muscarinic AChRs (mAChRs) [29]. The nAChRs belong to a well-defined family of ligand-gated ion channels that are widely expressed throughout ganglions and musculoskeletal junctions, which makes them important targets for the development of novel analgesics [30,31]. Nicotine activates nAChRs, especially the high-affinity α4β2 subtype and low-affinity α7 subtype in the central and peripheral nervous system, and even in non-neuronal cells to modulate pain signals transduction [32,33,34,35,36,37]. Long-term exposure to nicotine (tobacco) desensitizes the nAChRs in cholinergic synapses. When nAChRs expressed on GABA neurons are significantly desensitized, fewer nAChRs will be able to respond to acetylcholine released in the synapse, resulting in a decrease in GABAergic inhibition.

### 4.3. Strengths and Limitations

A noteworthy strength of our review lies in its multifaceted approach to examining the anti-nociceptive effects of nicotine in humans. We conducted meta-analytic assessments across various dimensions, including laboratory-induced pain threshold and pain tolerance in both healthy smokers and non-smokers. Additionally, we delved into a systemic review and meta-analysis of pain scores among patients, regardless of their nicotine intake, who were dealing with postoperative pain or various forms of chronic pain. This comprehensive methodology allowed us to provide a more thorough understanding of the intricate relationship between nicotine and pain.

Nonetheless, this systematic review and meta-analysis have several limitations that warrant acknowledgment. We confined our search to online databases in English, which raises the possibility of missing articles published in other languages or offline content. Furthermore, our exclusive inclusion of full-text articles may introduce bias by omitting unpublished research. It is worth noting that publication bias can potentially skew both between-group and within-group differences, as studies with significant or favorable results are more likely to be published. Nevertheless, it is important to emphasize that we diligently checked for publication bias to mitigate this potential limitation.

## 5. Conclusions

In conclusion, this study has demonstrated the analgesic effects of nicotine, particularly within the realm of acute pain. Patients who were administered nicotine via tobacco smoke exhibited pronounced analgesic responses to thermal stimuli in controlled laboratory-induced pain scenarios, consistent with prior research that has suggested short-term nicotine exposure’s capacity to elevate pain thresholds and enhance pain tolerance in controlled laboratory-induced pain scenarios. However, it is important to note that patients who consumed nicotine within 24 h following surgical procedures did not exhibit significant analgesic effects. This observation underscores the intricate and multifaceted nature of nicotine’s analgesic attributes, highlighting the necessity of considering various contextual factors. Moreover, this study delved into the ramifications of long-term nicotine usage. It revealed that chronic tobacco smoking may indeed contribute to hyperalgesia in the context of chronic pain conditions, underscoring the nuanced and multifaceted relationship between nicotine and pain management. These findings have raised legitimate concerns about the potential adverse consequences associated with chronic nicotine exposure, particularly regarding its potential to exacerbate chronic pain conditions. As for the analgesic effects of nicotine and other novel compounds acting on the same target, further animal experiments and multi-center clinical studies are needed.

## Figures and Tables

**Table 1:** Intervention, types of pain, outcome measures, study design, sample size, and quality assessment of studies included in this systemic review and meta-analysis.

Study	Year	Intervention	Pain	Measures	Design	Sample (T/C)	Quality Assessment (RoB2)					
							I1	I2	I3	I4	I5	Total
Zhu et al. [26]	2023	patch	surgery	NRS score	between	50/51	1	1	1	1	1	5
Landim et al. [17]	2020	patch	surgery	VAS score	between	20/20	1	1	1	1	1	5
Esmat et al. [15]	2016	patch	surgery	VAS score	between	20/20	1	1	0	1	1	4
Weingarten et al. [18]	2015	nasal spray	surgery	NRS score	between	42/47	1	1	1	1	1	5
Jankowski et al. [19]	2011	nasal spray	surgery	NRS score	between	72/79	1	1	1	1	1	5
Turan et al. [9]	2008	patch	surgery	VRS score	between	43/42	1	1	0	1	1	4
Hong et al. [16]	2008	patch	surgery	NRS score	between	30/10	1	1	0	1	1	4
Habib et al. [20]	2008	patch	surgery	VAS score	between	44/46	1	1	1	1	1	5
Flood et al. [8]	2004	nasal spray	surgery	NRS score	between	10/10	1	1	0	1	1	4
Mekhail et al. [22]	2020	tobacco	CRPS	NRS score	between	177/192	1	1	1	1	1	5
Lee et al. [23]	2020	tobacco	chronic	NRS score	between	164/60	1	0	1	1	1	4
Nastase et al. [10]	2007	tobacco	thermal	PTH and PTO	within	23/23	1	1	1	1	1	5
Kanarek et al. [11]	2004	tobacco	thermal	PTH and PTO	within	49/49	1	1	1	1	1	5
Lane et al. [12]	1995	tobacco	thermal	PTH and PTO	within	18/18	1	1	1	1	1	5
Fertig et al. [13]	1986	tobacco	thermal	PTH and PTO	within	10/10	1	1	0	1	1	4
Pomerleau et al. [14]	1984	tobacco	thermal	PTH and PTO	within	5/5	1	1	0	1	1	4

Quality assessment items. Item 1 (I1): bias arising from the randomization process; Item 2 (I2): bias due to deviations from intended interventions; Item 3 (I3): bias due to missing outcome data; Item 4 (I4): bias in measurement of the outcome; Item 5 (I5): bias in selection of the reported result. Abbreviations. T: nicotine group; C: control group; CRPS: complex regional pain syndrome; PTH: pain threshold; PTO: pain tolerance; VAS: visual analogue scale; NRS: numerical rating scale. Definitions (design column): between = randomized control comparison between groups; within = before–after study in the same patient group.

## Data Availability

Data are contained within the article and supplementary material.

## Supplementary Materials

The following supporting information can be downloaded at: https://www.mdpi.com/article/10.3390/ph16121665/s1, Figure S1: Funnel plots for publication bias removal. Each black dot represents a study. The X-axis represents the study effect size (hazard ratio) and the Y-axis represents the standard error of the hazard ratio. The dotted line indicates the overall risk estimate, and the black line indicates no intervention effect.

## Study Protocol Registration

The protocol of this systemic review and meta-analysis has been registered at https://www.crd.york.ac.uk/PROSPERO (accessed on 20 May 2021). The registration number is CRD42021250138. No significant amendments were made the protocol in the experimental design.
