# Supplementary material for: The Anti-Nociceptive Effects of Nicotine in Humans: A Systematic Review and Meta-Analysis
Source: Pharmaceuticals (Basel). 2023 Nov 30;16(12):1665. doi: 10.3390/ph16121665 (PMC10747127; doi:10.3390/ph16121665)
Supplement: Supplementary file 1 [file pharmaceuticals-16-01665-s001.zip › pharmaceuticals-2590933-supplementary.pdf]

Supplementary Materials: The anti-nociceptive effects of nicotine in humans: a systematic review and meta-analysis

Yujia Luo, Yating Yang, Carl Schneider and Thomas Balle

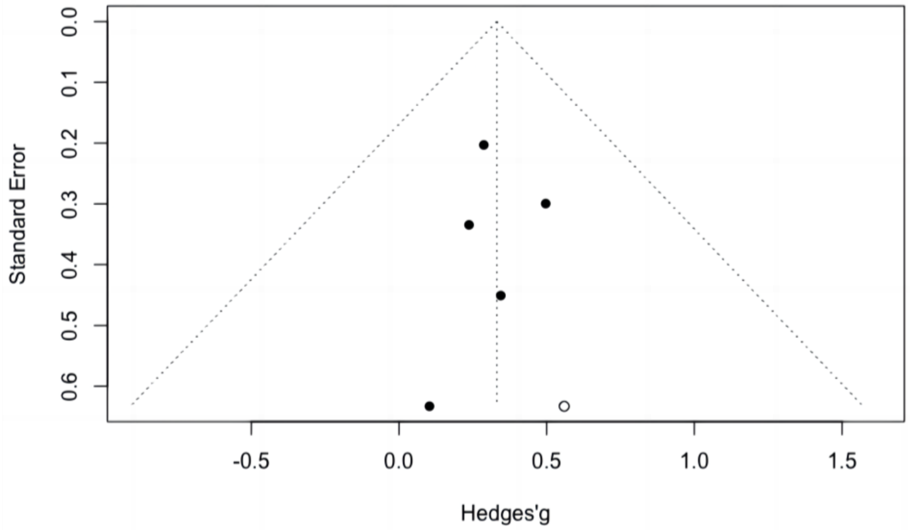

Figure S1. Funnel plots for publication bias removal. Each black dot represents a study. The X-axis represents the study effect size (hazard ratio) and the Y-axis represents the standard error of the hazard ratio. The dotted line indicates the overall risk estimate, and the black line indicates no intervention effect.
